# Molecular characterization of *Arcobacter butzleri* isolates from poultry in rural Ghana

**DOI:** 10.3389/fcimb.2023.1094067

**Published:** 2023-01-25

**Authors:** Andreas E. Zautner, Thomas Riedel, Boyke Bunk, Cathrin Spröer, Kennedy G. Boahen, Charity Wiafe Akenten, Annika Dreyer, Jacqueline Färber, Achim J. Kaasch, Jörg Overmann, Jürgen May, Denise Dekker

**Affiliations:** ^1^ Institut für Medizinische Mikrobiologie und Krankenhaushygiene, Universitätsklinikum Magdeburg, Magdeburg, Germany; ^2^ Abteilung Mikrobielle Ökologie und Diversitätsforschung, Leibniz-Institut DSMZ-Deutsche Sammlung von Mikroorganismen und Zellkulturen GmbH, Braunschweig, Germany; ^3^ Deutsches Zentrum für Infektionsforschung (DZIF), Hannover–Braunschweig, Germany; ^4^ Abteilung Bioinformatik und Datenbanken, Leibniz-Institut DSMZ-Deutsche Sammlung von Mikroorganismen und Zellkulturen GmbH, Braunschweig, Germany; ^5^ One Health Bacteriology Group, Kumasi Centre for Collaborative Research in Tropical Medicine (KCCR), Kumasi, Ghana; ^6^ Institut für Medizinische Mikrobiologie und Virology, Universitätsmedizin Göttingen, Göttingen, Germany; ^7^ Abteilung für Infektionsepidemiologie, Bernhard-Nocht-Institut für Tropenmedizin (BNITM), Hamburg, Germany; ^8^ Deutsches Zentrum für Infektionsforschung (DZIF), Hamburg-Borstel-Lübeck, Germany

**Keywords:** *Arcobacter butzleri*, poultry, antimicrobial susceptibility, genome, Africa, Ghana

## Abstract

In recent years, *Arcobacter butzleri* has gained clinical significance as an emerging diarrheagenic pathogen associated with poultry and water reservoirs. The full clinical significance of *Arcobacter* remains rather speculative due to variable virulence and antibiotic susceptibility of individual strains. The aims of the present study were (i) to identify antibiotic resistance genes (ARGs) in the genome sequences of two multidrug-resistant *A. butzleri* isolates, (ii) to use multilocus-sequence typing (MLST) to generate a guiding phylogeny of *A. butzleri* isolates collected in Kumasi, Ghana, (iii) to examine the distribution of ARGs in the test cohort, and (iv) to assess the strain’s virulence and possible antibiotic treatment options for arcobacteriosis based on the genome sequences and the ARG distribution. A total of 48 A*. butzleri* isolates obtained from poultry were included in the analysis. These isolates were genotyped by MLST and the antibiotic susceptibilities of isolates to ampicillin, ciprofloxacin, tetracycline, gentamicin, and erythromycin were tested by disk diffusion. Whole genome sequence data of two multidrug-resistant (MDR) *A. butzleri* isolates were obtained by a combination of single-molecule real-time (SMRT) and Illumina sequencing technology. A total of 14 ARGs were identified in the two generated genome sequences. For all 48 isolates, the frequency of these 14 ARGs was investigated by PCR or amplicon sequencing. With 44 different sequence types found among 48 isolates, strains were phylogenetically heterogeneous. Four of 48 isolates showed an ARG constellation indicating a multidrug-resistant phenotype. The virulence genes in the two *A. butzleri* genomes showed that the species might be characterized by a somewhat lower virulence as *Campylobacter* species. The phenotypic susceptibility data combined with the distribution of the particular ARGs especially *oxa-464* and the T81I point mutation of the quinolone resistance determining region (QRDR) in a significant percentage of isolates indicated that macrolides and tetracycline can be recommended for calculated antibiotic treatment of arcobacteriosis in Ghana, but not ampicillin and quinolones.

## 1 Introduction

During the last years, *Arcobacter* spp. (synonym: *Aliarcobacter* spp.) have gained clinical importance as emerging diarrheagenic pathogens associated with food products, especially those of animal origin, or with water (Kabeya et al., 2004; Scullion et al., 2006; Alonso et al., 2014). Of the 33 known *Arcobacter* species, *A. butzleri* and *A. cryaerophilus* have been recognized as the most important species causing human enteritis (Ferreira et al., 2016). Data on transmission of *Arcobacter*, on the frequencies of infections caused and their distribution is generally limited. Its significance as an important pathogen causing human infections worldwide might therefore be underestimated. This is particularly true for sub-Saharan African countries where diagnostic resources are limited and *Arcobacter* is not routinely cultured and antibiotic susceptibility testing not performed. Despite these information gaps, poultry has been recognized as major reservoir and vehicle for transmissions (Ferreira et al., 2016).

In Africa, *Arcobacter* spp. have been isolated from poultry in Egypt (Hassan, 2017), from poultry slaughterhouse effluent waste and live poultry in Nigeria (Amisu et al., 2003; Adesiji et al., 2011) as well as chicken meat in Tunisia (Jribi et al., 2017). *Arcobacter* has also been detected in approximately 11% of diarrhea patients in South Africa by PCR (Samie et al., 2007). Regrettably, *Arcobacter* is not routinely investigated in stool in Ghana and thus its prevalence in either human or animal sources in this country is largely unknown.

Fluoroquinolones, tetracyclines and macrolides are the first line treatment options for severe infections with *Arcobacter* (Son et al., 2007). While a low resistance rate to fluoroquinolones has been observed in most countries, a comparatively high resistance rate has been reported for Portugal and Costa Rica (Ferreira et al., 2013; Villalobos et al., 2013). So far, Thr-85-Ile is the only known mutation in the *gyrA* gene capable of inducing ciprofloxacin resistance in *Arcobacter* spp. (Ferreira et al., 2018). Multidrug resistance in *Arcobacter* is scarcely reported but sporadic reduced susceptibility to antibiotics has been observed by several studies (Ferreira et al., 2013; Millar and Raghavan, 2017; Šilha et al., 2017; Isidro et al., 2020; Jribi et al., 2020; Jasim et al., 2021; Sciortino et al., 2021).

Multilocus sequence typing (MLST) and whole genome sequencing (WGS) have been employed to provide more robust data including detailed molecular characteristics and phylogenetical linkages for epidemiological purposes. Miller’s pioneering work using MLST for *Arcobacter* has been recognized as the gold standard for analyses (Merga et al., 2013). Since the MLST data in the *Arcobacter* pubMLST database (https://pubmlst.org/organisms/arcobacter-spp) are so far limited, unreported allele profiles are detected frequently. This hinders an adequate identification and evaluation of clinically important sequence types (STs) (Merga et al., 2013). MLST as an epidemiological tool offers adequate discriminatory power for strain differentiation and analysis. Only few studies have applied MLST on isolates from poultry and to our knowledge this method has not been applied to any African isolates.

Evidence for variations in survival and sensing systems which may enhance the adaptation to different hosts have been detected using WGS in *A. butzleri* (Merga et al., 2013). Genome sequencing data of *A. butzleri* from human infections (Hume et al., 2001), microbial fuel cells (Toh et al., 2011) and cattle (Merga et al., 2013) have been obtained. However, WGS has not been used before to explore strains isolated from poultry in Africa.

The aim of this investigation was to use MLST and WGS for the assessment of the diversity and molecular characteristics of *A. butzleri* including two multidrug-resistant strains isolated from poultry meat in Ghana. Furthermore, the antibiotic resistance genes (ARGs) were identified in the genome sequences of the two multidrug-resistant *A. butzleri* isolates and their distribution in the MLS-typed isolate cohort was investigated by PCR. The genome sequences were also analyzed to assess the pathogenic potential of the MDR isolates based on the genes encoding presumptive virulence-associated factors. Previously, Miller and colleagues described the genome of a non-multidrug resistant *A. butzleri* strain (RM4018), differing from the two *A. butzleri* genomes analyzed within this study in many aspects, just because of the antibiotic resistance situation. In addition to the identification of resistance genes, we aimed at identifying plasmids, virulence-associated genes, prophages and surface structures that could potentially cause postinfectious sequelae *via* possible molecular mimicry.

## 2 Materials and methods

### 2.1 Study site and sample collection

Between May and December 2015, chicken meat was collected in Kumasi, the capital of the Ashanti region of Ghana. A total of 75 supermarkets were visited for the collection of frozen imported chicken meat. The country of origin was retrieved from the packaging. When meat from different countries was available in the same supermarket, one from each country was collected. In addition, for local meat, freshly slaughtered chicken meat was collected from 19 retailers. For each sample, 15 g of chicken meat was collected in a sterile plastic bag and transported in a cool box at 2-8°C to the laboratory within 2-4 h.

### 2.2 Bacterial detection and identification

The meat was sliced with a sterile scalpel in a petri dish and incubated for 18-24 h at 30°C in 10 mL *Arcobacter* enrichment broth (Oxoid, Basingstoke, England) under microaerophilic conditions (CampyGen sachets, Oxoid). Bacterial cells from the *Arcobacter* enrichment broth were subsequently cultured on Mueller Hinton agar (Oxoid) with 5% (vol/vol) sheep blood using a filter technique as described by Atabay et al. (Atabay et al., 2003). The plates were incubated at 35–37°C for 18–24 h under microaerophilic conditions. Plates showing no sign of bacterial growth were incubated for four additional days. The identity of oxidase-positive isolates was assessed by Matrix Assisted Laser Desorption Ionization Time of Flight mass spectrometry (MALDI-TOF MS; Bruker Daltonics GmbH & Co. KG, Bremen, Germany).

### 2.3 Antibiotic susceptibility testing

Antibiotic susceptibility was tested by the disk diffusion method (Kirby Bauer). Antibiotic test disks were placed on agar plates with Mueller Hinton agar supplemented with 5% horse blood (MHF) inoculated with an *A. butzleri* bacterial suspension (MacFarland: 0.5). The test agar plates were incubated at 42°C under microaerophilic conditions for 24 h. The microaerophilic atmosphere was generated using a Whitley jar gassing system (Don Whitley Scientific Limited, Bingley, UK). Bacterial isolates with insufficient growth after 24 h were re-incubated, and the inhibition zone was measured after a total of 40–48 h. Since there are no breakpoints defined for *Arcobacter* neither according to the European Committee on Antimicrobial Susceptibility Testing (EUCAST) nor the Clinical & Laboratory Standards Institute (CLSI), the zone diameter was simply measured and an epidemiological cut-off (Ecoff) value determined on the basis of all tested isolates (for the definition of Ecoff values, see results). Isolates were tested for tetracycline (TE30), ciprofloxacin (CIP5), erythromycin (E15), gentamicin (CN10), and ampicillin (AMP10). Antimicrobial test discs were obtained from Oxoid/Thermo Fisher Scientific (Wesel, Germany). The laboratory work was carried out in a laboratory accredited according to DIN EN ISO 15189. The quality control strains used for resistance testing by agar diffusion were *Campylobacter jejuni* ATCC 33560 for the antibiotic test discs CIP5 (5 µg ciprofloxacin per disc), E15 (15 µg erythromycin per disc), and TE30 (30 µg tetracycline per disc), *Pseudomonas aeruginosa* ATCC 27853 for CN10 (10 µg gentamicin per disc), and *Enterococcus faecalis* ATCC 29212 for AMP10 (10 µg ampicillin per disc). Resistance to the three classes of antibiotics comprised of tetracyclines, fluoroquinolones, and macrolides was defined as multidrug resistance (MDR).

### 2.4 Genome sequencing

High molecular weight DNA was prepared using Qiagen Genomic Tip/100 G (Qiagen, Hilden, Germany) according to the instructions of the manufacturer. SMRTbell™ template library was prepared according to the instructions from Pacific Biosciences, Menlo Park, CA, USA, following the Procedure & Checklist - 10 kb or 20 kb Template Preparation Using BluePippin™ Size-Selection System. SMRT sequencing was carried out on the PacBio *RS II* or Sequel II (Pacific Biosciences, Menlo Park, CA, USA). 1 SMRT Cell (on the PacBio *RS II*) was used to complete the *A. butzleri* P1200 genome; 2 SMRT Cells (one SMRT Cell on the PacBio *RS II*, and one SMRT Cell on the PacBio Sequel II) were necessary to sequence the *A. butzleri* P1100 genome. Short read sequencing was performed from the same DNA preparation on the Nextseq (llumina Inc., San Diego, CA, USA).

### 2.5 Genome assembly and bioinformatics analysis

Genomes were assembled independently using the `RS_HGAP_Assembly.3´ protocol included in SMRT Portal version 2.3.0 using default parameters. Since the genome sequence of strain *A. butzleri* P1100 could not be assembled completely, raw data from the PacBio *RSII* were downloaded as filtered subreads (filtered_subreads.fastq) *via* the SMRTPortal 2.3.0. Additionally, raw BAM data from the PacBio Sequel II were converted to fastq using the “Convert BAM to FASTX” application included in SMRTlink 10. Afterwards, a combined genome assembly of both datasets has been performed using CANU 2.0 applying a target genome size of 3 Mbp and specifying both fastq.(gz) files as PacBio raw data input (Koren et al., 2017). For the PacBio long-read assembly of strain *A. butzleri* P1200, 66,165 postfiltered reads with an average read length of 12,740 bp were used. Replicons were trimmed and circularized. The chromosomal contig was adjusted to *dnaA* (chromosomal replication initiation protein DnaA) and the plasmid to *soj* (sporulation initiation inhibitor protein soj). The validity of the assembly was checked using the `RS_Bridgemapper.1´ protocol.

Finally, each genome was corrected for InDel errors by a mapping of Illumina reads onto finished genomes using the Burrows-Wheeler Alignment tool (BWA) (Li and Durbin, 2009) with subsequent variant and consensus calling using VarScan 2 (Koboldt et al., 2012). A consensus concordance of QV60 could be confirmed for both genomes. Finally, automated genome annotation was generated using Prokka (Seemann, 2014). Checking for acquired ARGs was performed using ResFinder 3.2 available at the Center for Genomic Epidemiology (https://cge.cbs.dtu.dk/services/ResFinder/) (Camacho et al., 2009; Zankari et al., 2017; Bortolaia et al., 2020). The rapid annotation using subsystem technology platform (RAST, http://rast.nmpdr.org) was consulted to identify functional subsystems in the *A. butzleri* genomes (https://rast.nmpdr.org/) (Aziz et al., 2008; Overbeek et al., 2014; Brettin et al., 2015). CRISPRCasFinder (https://crisprcas.i2bc.paris-saclay.fr/) was used to check both genome sequences for clustered regularly interspaced short palindromic repeats (CRISPRs) (Kurtz and Schleiermacher, 1999; Abouelhoda et al., 2004; Hyatt et al., 2010; Abby et al., 2014; Biswas et al., 2014). The presence of prophages in the genomes was assessed using the PHAge Search Tool – PHASTER (Arndt et al., 2016). Visualization of chromosome/genome comparison was performed using the Blast Ring Image Generator (BRIG) (Alikhan et al., 2011).

### 2.6 Multilocus sequence typing

For the MLST-typing of the *A. butzleri* isolates, the original method was used previously described by Miller et al., 2009 (Miller et al., 2009). Accordingly, *A. butzleri* specific primers (https://pubmlst.org/arcobacter/) were used to amplify the seven housekeeping genes used in the MLST scheme. The *lysS*-linked *glyA1* allele was amplified for typing (Miller et al., 2009). The MEGA X software for Debian/Ubuntu-based Linux distributions programmed by Kumar et al. (Kumar et al., 2018) was used to construct an MLST-based UPGMA (unweighted pair group method with arithmetic mean) dendrogram (Sneath and Sokal, 1973) after concatenating of the MLST gene sequences for each strain.

### 2.7 Assessment of ARG distribution and QRDR sequencing

The distribution of the ARGs in the *A. butzleri* isolate collection was investigated by PCR using the primers listed in **Table 1**. PCR-reactions were conducted with the following parameters: two denaturation steps at 95°C for 30 s; annealing at the annealing temperature listed in **Table 1** for 1 min; followed by two elongation steps at 68°C for 1 min and 5 min. The quinolone resistance-determining region (QRDR) of the *gyrA* gene was sequenced according to the method of Abdelbaqi and colleagues (Abdelbaqi et al., 2007). Sanger sequencing of the QRDR amplificons was performed employing the ExoSAP-IT™ and BigDye Terminator v3.1 Cycle Sequencing kit (Applied Biosystems by Thermo Fisher Scientific, Vilnius, Lithuania.) on the ABI PRISM 3130xl Genetic Analyzer (Applied Biosystems, Foster City, USA).

**Table 1:** Primers used for amplification of *A. butzleri* antibiotic resistance genes.

gene	primer name	sequence 5’-3’	annealing temperature	amplicon length
quinolone resistance determining region (QRDR) (Abdelbaqi et al., 2007)	F–QRDR	TGGATTAAAGCCAGTTCATAGAAG	58°C	344 bp
	R2–QRDR	TCATMGWATCATCATAATTTGGWAC		
*tetA* tetracycline resistance (tetH)	ABu-tetA-F01	TCACTGGCAACCCATTACGG	59°C	260 bp
	ABu-tetA-R01	GCGGGAGTCACATCACTCAT		
*tetM* tetracycline resistance	ABu-tetM-F01	AGCTCATGTTGATGCGGGAA	59°C	728 bp
	ABu-tetM-R01	TTCCGCAAAGTTCAGACGGA		
*tetL* gene for tetracycline efflux MFS transporter TetL	Abu-tetL-F01	TTCGGGTCGGTAATTGGGTT	59°C	828 bp
	Abu-tetL-R01	TTTGGTGAACGAAAGCCCAC		
*bla*_OXA-464_ beta-lactamase	Abu-OXA-464-F01	CATCGTTTTCTCCTGCTTCAACA	59°C	447 bp
	Abu-OXA-464-R01	TTCCTTCCCAACCTGTTTTTGC		
*macA* macrolide resistance	Abu-macA-F01	CAGCAACTATGTCTGCTCCAAA	58°C	417 bp
	Abu-macA-R01	CTCTTGCAACATTTGCAGGTCT		
*macB* macrolide resistance	Abu-macB-F01	GAAGAGGACCACCAAGAGAAGA	59°C	396 bp
	Abu-macB-R01	CTTGGTCATTCCCAAAAGCAGC		
*tolC* Type I secretion outer membrane protein	Abu-tolC-F01	TCAGGTTTATCACAAACTCACTCT	58°C	935 bp
	Abu-tolC-R01	ACCGAAGTCATTCCTGCTTCA		
*ermB* 23S rRNA (adenine(2058)-N(6))-methyltransferase ErmB	Abu-ermB-F01	ACTAGGGTTGCTCTTGCACA	59°C	125 bp
	Abu-ermB-R01	CATCTGTGGTATGGCGGGTA		
*str* Streptomycin adenylyltransferase Str	Abu-Str-F02	TGAGTTTTGGAGTGTCTCAACG	57°C	423 bp
	Abu-Str-R02	AATCAAAACCCCTATTAAAGCCAA		
*cat* chloramphenicol *O*-acetyltransferase Cat (pC194)	Abu-cat-F01	GGTTACAATAGCGACGGAGAGT	58°C	426 bp
	Abu-cat-R01	AGGCCTATCTGACAATTCCTGA		
*ant(6)-Ia* aminoglycoside nucleotidyltransferase ANT(6)-Ia	Abu-ant6I-F01	ATGATGCAAAAGCCGGAGGA	60°C	388 bp
	Abu-ant6I-R01	CGATGCCGACCTTCCATGAT		
*aph(3)-IIIa* 3-aminoglycoside *O*-phosphotransferase type IIIa Aph(3)-IIIa	Abu-aphIIIa-F01	ATCGAGCTGTATGCGGAGTG	60°C	328 bp
	Abu-aphIIIa-R01	TGTCATACCACTTGTCCGCC		
*aadD* aminoglycoside adenyltransferase AadD/kanamycin nucleotidyltransferase	Abu-aadD-F01	GCAGGTGCCATGTTGATTGG	60°C	214 bp
	Abu-aadD-R01	ATCCGTGTCGTTCTGTCCAC		

## 3 Results and discussion

### 3.1 Multilocus sequence typing

**Figure 1:**
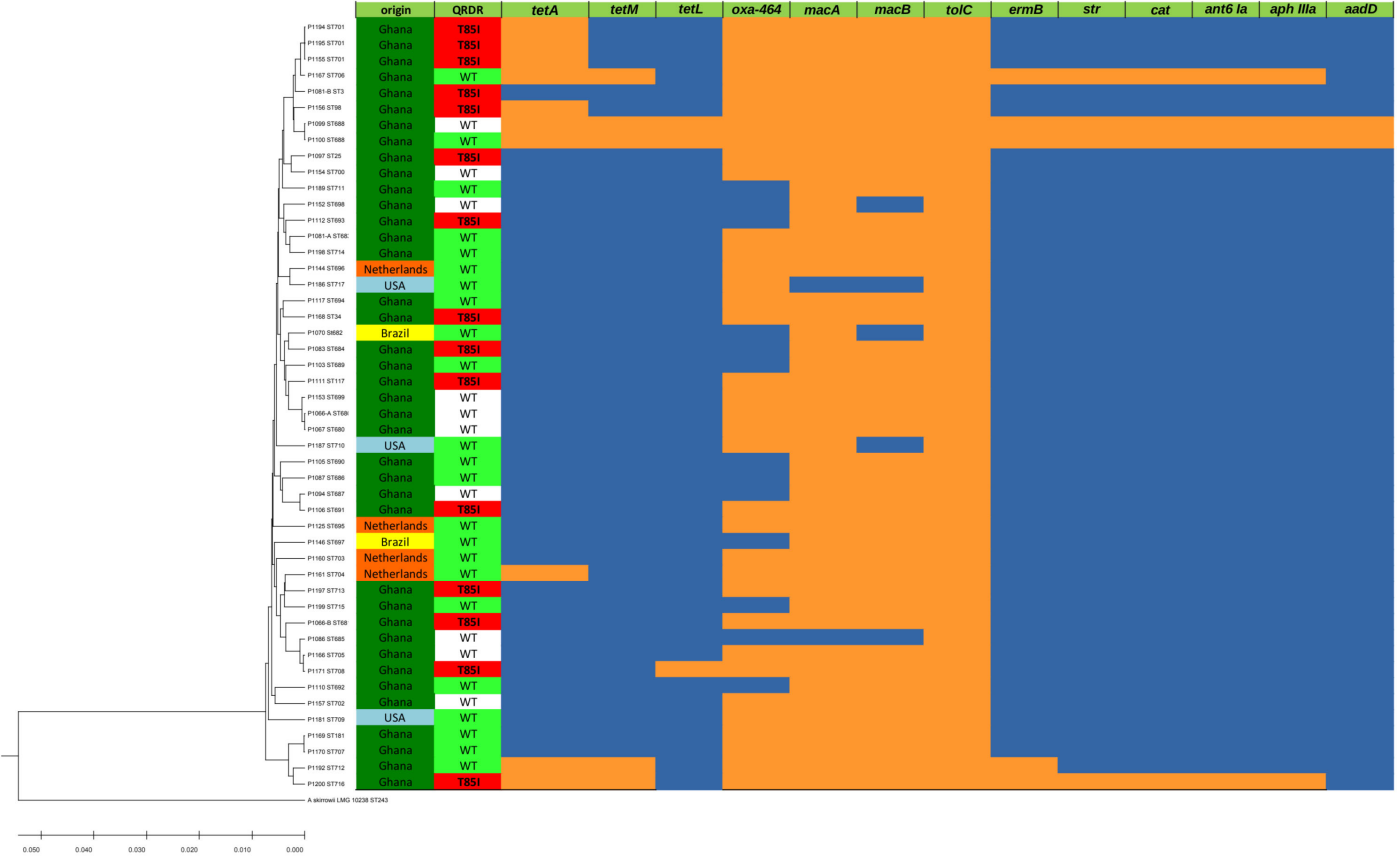
MLST-based UPGMA-tree and heatmap of ARGs. Left side: MLST-based UPGMA-dendrogram: The evolutionary history of the 48 *A. butzleri* isolates was inferred using the UPGMA method. For reference *A. skirrowii* LMG 10238 was included as outgroup in our analysis. The depicted optimal tree is drawn to scale and has a sum of branch length of 0.27052614, with branch lengths in the same units as those of the evolutionary distances used to infer the phylogenetic tree. Right side: Legend/Color codes: origin: green – Ghana, orange – The Netherlands, light blue – USA, yellow – Brazil; QRDR (quinolone resistance determining region): red – T85I amino acid substitution in the QRDR associated with ciprofloxacin resistance (inhibition zone diameter < 13 mm), green – wild type (WT) QRDR and phenotypic ciprofloxacin susceptible isolate (inhibition zone diameter > 19 mm), white – wild type (WT) QRDR but phenotypic ciprofloxacin resistant (< 13 mm) isolate; ARGs (see also Table 1): orange – positive PCR result, blue – negative PCR result.

A total of 48 *A. butzleri* isolates were analyzed using MLST. These included four isolates from meat imported from the Netherlands, three from meat imported from the USA, two from meat imported from Brazil, and 39 from poultry produced locally in Ghana. MLST-typing of these 48 *A. butzleri* isolates resulted in 44 different sequence types (STs). Therefore, the isolated *A. butzleri* test population was diverse and clonalities were not found. The resulting MLST-based UPGMA dendrogram is shown in **Figure 1**. Regarding the origin of the broiler carcasses, a small cluster of three Dutch isolates was detected among the isolates. The other isolates from abroad show no clustering, i.e., two isolates from Brazil and three isolates from the USA, as well as another isolate from the Netherlands mix phylogenetically with the local isolates from Ghana. Of the 44 STs, 42 STs were not previously described and therefore submitted to the Pubmlst database as new dataset.

### 3.2 Genome sequencing and annotation

Based on susceptibility testing results, we selected two *A. butzleri* isolates resistant to four of the five antibiotics tested (all isolates tested were gentamicin susceptible), that showed a vast MLST-based phylogenetic distance, for SMRT sequencing, at the time under the assumption that they were multidrug-resistant isolates. These two isolates were *A. butzleri* P1100 belonging to ST688 and *A. butzleri* P1200 belonging to ST716. Both isolates were isolated from chicken originating from Ghana. The genome assemblies yielded a circular chromosome for both strains and two extrachromosomal elements (plasmids) in P1100 and one in P1200.

The *A. butzleri* P1100 genome consisted of a single contig of 2,367,006 bp, the bacterial chromosome, the second contig represented a plasmid of a length of 63,921 bp, and the third contig represented a plasmid of a length of 27,880 bp. The GC content of this contig (chromosome) was 27.18%. Utilization of the NCBI annotation pipeline resulted in 2,553 predicted genes encoded on the genome, of these 2,465 were presumably protein-coding genes, 17 were pseudogenes, and 71 were RNA-coding genes. Of the 71 presumptive RNA-coding genes, 54 encoded tRNAs, two encoded ncRNAs, five encoded 5S rRNAs, five encoded 16S rRNAs, and five encoded 23S rRNAs. The *A. butzleri* P1100 genome harbored neither prophages nor transposable elements. The RASTtk subsystem coverage was 25% (619 genes) and therewith relatively low. Of these subsystems amino acid metabolism (178 of 841 associated terms, 21.2%), protein metabolism (119 of 841 terms, 14.1%), cofactors, vitamins, prosthetic groups, and pigments (95 of 841 terms; 11.3%), and respiration (61 of 841 terms; 7.3%) represented the largest functional groups.

**Figure 2:**
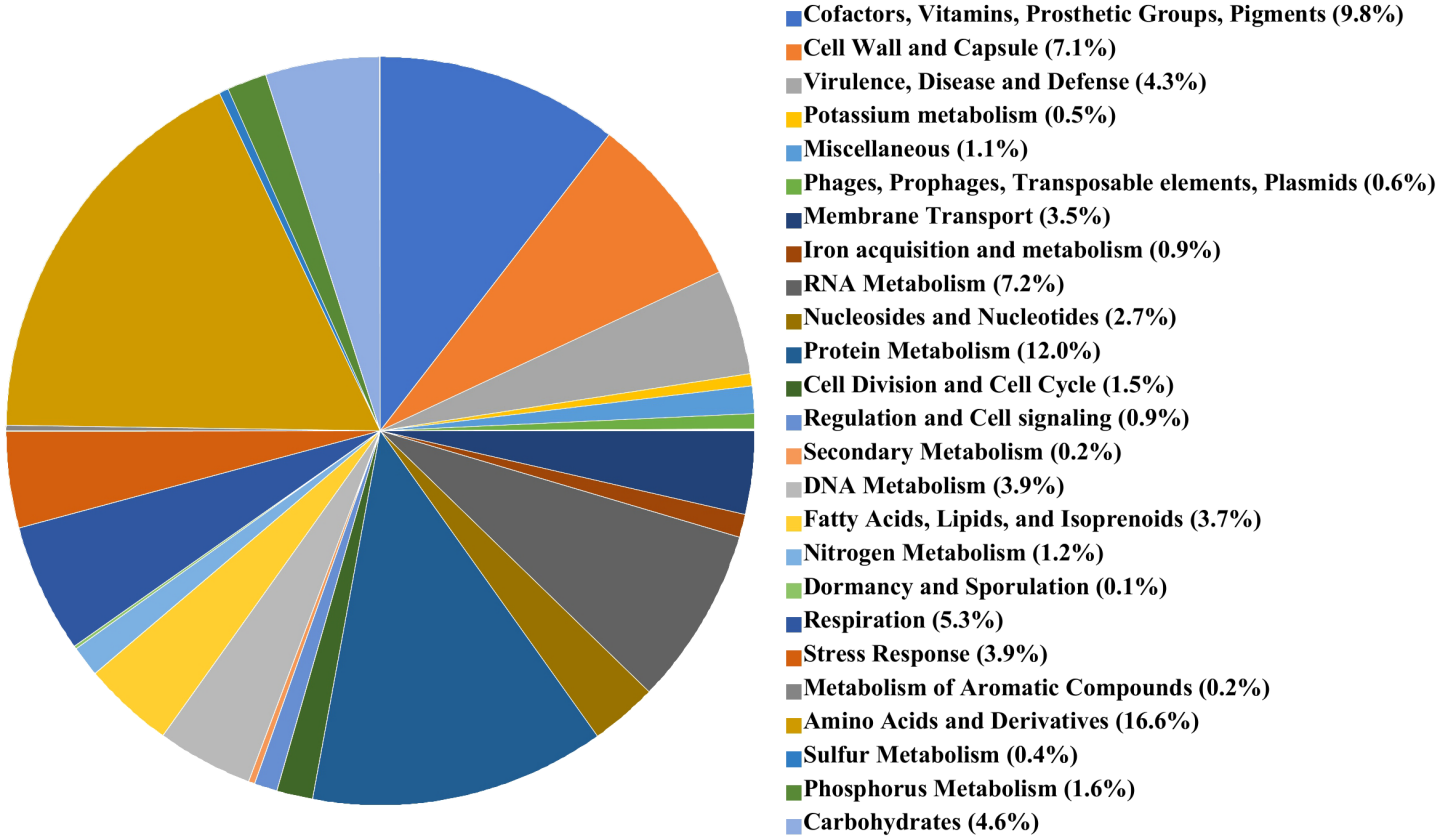
RAST subsystems in *A. butzleri* P1200 represented as pie chart. The 25 most abundant subsystems identified by the SEED Viewer version 2.0 are represented by a specific color as indicated in the color legend at the right side.

The genome of *A. butzleri* P1200 consisted of a single contig of 2,143,012 bp, the bacterial chromosome, and a second contig representing a plasmid of a length of 83,142 bp. The GC content of the P1200 chromosome was 27.16%. Application of the NCBI-annotation pipeline onto the *A. butzleri* P1200 genome resulted in 2,269 genes. Of this total number of genes, 2,183 were protein-coding genes, 15 were pseudogenes, and 71 were RNA-coding genes. Of the 71 RNA-coding genes, 54 encoded tRNAs, two encoded ncRNAs, five encoded 5S rRNAs, five encoded 16S rRNAs, and five encoded 23S rRNAs. The *A. butzleri* P1200 genome harbored neither prophages nor transposable elements. The RASTtk subsystem coverage of *A. butzleri* P1200 was with 50% and thus significantly higher than for *A. butzleri* P1100 and more representative of the microbial species *A. butzleri*. Of these subsystems, amino acid metabolism (269 of 1618 associated terms, 16.6%), cofactors, vitamins, prosthetic groups, and pigments (195 of 1618 terms; 12.1%), and protein metabolism (194 of 1618 terms, 12.0%), again represented the largest functional categories. These were followed by RNA metabolism (117 of 1618 associated terms, 7.2%), cell wall and capsule synthesis (115 of 1618 associated terms, 7.1%), and motility and chemotaxis (97 of 1618 associated terms, 6.0%). A pie chart illustrating subsystem category distribution in *A. butzleri* P1200 is shown in **Figure 2**.

**Figure 3:**
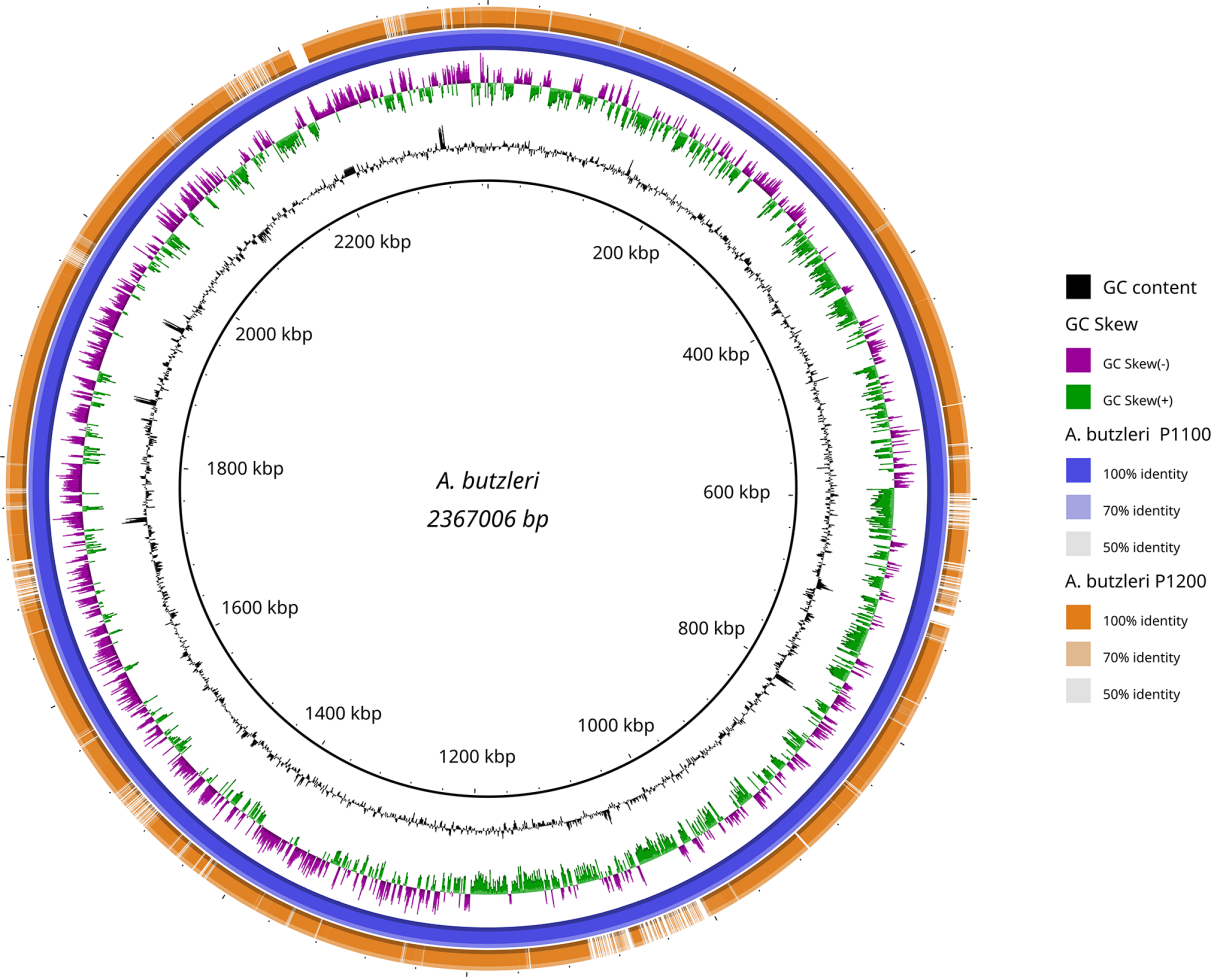
Blast Ring Image Generator (BRIG) generated graph depicting homologous chromosome segments of *A. butzleri* P1100 (reference) and P1200. *A. butzleri* P1100 was chosen as reference (inner black circle) and is additionally shown in the second outer circle (blue). The outermost orange ring shows the homology of *A. butzleri* P1200 compared to *A. butzleri* P1100 based on BLASTn+ analysis. Additionally GC content (black) and genomic GC skew (purple/green) are also shown in the figure.

A rough comparison of both genomes shows that the genome of *A. butzleri* P1100 is 232,653 bp larger than that of *A. butzleri* P1200. The additional genes in the genome of *A. butzleri* P1100 compared to *A. butzleri* P1200 cluster in about 7 regions (**Figure 3**). The GC content is similar in both genomes at nearly 27.2%, but is significantly lower compared to *C. jejuni* at 30.5% (NCTC 11168) (Parkhill et al., 2000). This indicates a lower number of coding regions per genomic region (of a comparably large arbitrary length) in comparison to *C. jejuni*. As indicated in **Figure 3** the GC skew of the *A. butzleri* genome was predominantly positive in the region 300,000 bp – 1,300,000 bp but predominantly negative in the region 1,300,000 bp – 300,000 bp indicating a switch of the leading strand.

### 3.3 Extrachromosomal elements (Plasmids) and mobile genetic elements (MGEs)

**Figure 4:**
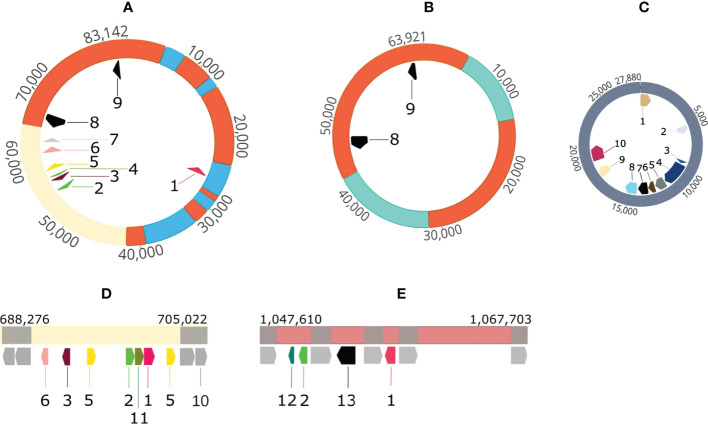
Visualization of plasmids and ARG-carrying mobile genetic elements in *A. butzleri* P1100 and P1200. **(A)** P1200 plasmid 83 kb 1 streptomycin 3’’-*O*-adenylyltransferase/spectinomycin 9-*O*-adenylyltransferase gene, 2: aminoglycoside 6-adenylyltansferase gene, 3: chloramphenicol acetyltransferase gene, 4: aminoglycoside 6-adenylyltransferase gene, 5: *ermB* gene for 23S rRNA (adenine(2058)-*N*(6))-methyltransferase Erm(B)- RRNA (Adenine-*N*(6)-) -methyltransferase, 6: *ant*(6)-Ia aminoglycoside nucleotidyltransferase ANT(6)-Ia gene, 7: *aph*(3)-IIIa 3-aminoglycoside *O*-phosphotransferase type IIIa Aph(3)-IIIa gene, 8: tetracycline resistance protein gene *tetM*, 9: tetracycline resistance MFS efflux pump gene *tetH*; **(B)** P1100 plasmid 63 kb 8: tetracycline resistance protein gene *tetM*, 9: tetracycline resistance MFS efflux pump gene *tetH*; **(C)** P1100 pVir-like plasmid 27 kb 1: plasmid partitioning protein gene *parB*, 2: *mpr4*, zinc metalloproteinase Mpr protein gene, 3: *virB2*, 4: *virB4*, 5: *virB6*, 6: *virB8*, inner membrane protein forms channel for type IV secretion of T-DNA complex, 7: *virB9* outer membrane and periplasm component of type IV secretion of T-DNA complex, 8: *virB10* inner membrane protein of type IV secretion of T-DNA complex, 9: *virB11* 10: *virD4*, ATPase required for T-DNA transfer; **(D)** P1100 1^st^ mobile genetic element 1: streptomycin 3’’-*O*-adenylyltransferase/spectinomycin 9-*O*-adenylyltransferase gene, 2: aminoglycoside 6-adenylyltansferase gene, 3: chloramphenicol acetyltransferase gene, 5: *ermB* gene for 23S rRNA (adenine(2058)-*N*(6))-methyltransferase Erm (B)- RRNA (Adenine-*N*(6)-) -methyltransferase, 6: *ant*(6)-Ia aminoglycoside nucleotidyltransferase ANT(6)-Ia gene, 10: mobile element protein gene, 11: *sat-4* streptothricin acetyltransferase gene; **(E)** P1100 2^nd^ mobile genetic element 1: streptomycin 3’’-*O*-adenylyltransferase/spectinomycin 9-*O*-adenylyltransferase gene, 2: aminoglycoside 6-adenylyltansferase gene, 12: *ble* bleomycin resistance protein gene, 13: tetracycline resistance MFS efflux pump gene *tetL;* Color codes: orange: sequence identical regions of P1200-plasmid 83 kb and P1100 plasmid 63 kb, cream colored: sequence identical regions of P1200-plasmid 83 kb and P1100 1^st^ mobile genetic element grey: mobile element protein genes other colors: indicate unique sequence segments.

In contrast to the reference strain *A. butzleri* RM4018, *A. butzleri* P1200 harbored one plasmid (p83_DSM_105473) with a size of 83,142 bp (**Figure 4A**, for comprehension reasons we start with the largest plasmid). Located near the origin of replication of the plasmid were the genes for the plasmid partitioning proteins ParA and ParB. The plasmid contained a total of nine ARGs (**Figure 4A**). This means that besides *macA*, *macB*, *tolC*, *bla*_OXA-464_ and *tetL* (only in P1100) all other ARGs were located on this plasmid. The plasmid also contained genes that encode thioredoxin, methyl-accepting chemotaxis protein I (serine chemoreceptor protein) and chaperone protein HtpG.

*A. butzleri* P1100 harbored two plasmids. The first plasmid had a size of 63,921 bp (**Figure 4B**) and carried the two tetracycline resistance genes *tetM* and *tetH*. A Mauve alignment showed that this 63.9 kb plasmid shared two major sequence segments with the 83.1 kb plasmid of P1200. These were the segments from 42,932 to 5,255 (circularized plasmid) and 14,122 to 31,388 (inverse orientation). Overall, there was approximately 68% coverage of the 83.1 kb resistance plasmid of P1200. In the *A. butzleri* P1200 resistance plasmid, the 48.187 bp segment corresponded to a total of three plasmid segments and the 17,266 bp segment to a total of four plasmid segments (**Figures 4A, B**). Both resistance plasmids of the two *A. butzleri* isolates sequenced in this study exhibit a high degree of evolutionary relatedness to each other. A BLAST search in the NCBI database revealed a maximum coverage of 15% and 33%, respectively, to plasmid pM830MA of *Arcobacter cryaerophilus* M830MA, so both plasmids were described here for the first time.

Furthermore, we were able to identify two mobile genetic elements (MGEs) in the genome of *A. butzleri* P1100 (**Figures 4D, E**). The first MGE was 16,756 bp in size and contained a region with high sequence identity to the region from 53,164 bp to 64,728 bp of the resistance plasmid of *A. butzleri* P1200. It haboured the resistance genes *ant*(6)-I, *catA*, *ermB* (two copies), *ant*(6)-I, *sat*-4, *aph*(3’)-III/*aph*(3’)-IV. The second MGE (**Figure 4E**) had a size of 20,093 bp and exhibited no regions of high sequence identity to the resistance plasmid of *A. butzleri* 1200. However, the four resistance genes *ble*, *ant*(4’)-Ia, *tetL*, and *aadA* were present on this MGE.

The second plasmid in the *A. butzleri* P1100 genome had a size of 27,880 bp. This 27.9 kb plasmid showed 94.4% sequence identity with a query coverage of 88% to the plasmid AB-1119-LD (GenBank accession: KF740630, length 27,476 bp) of *A. butzleri* AC1119 (Douidah et al., 2014). This plasmid carries the genetic information for a type IV secretion system that plays a role in T-DNA transfer. This shows functional similarities to the pVir plasmid of *C. jejuni* (Bacon et al., 2002). The 27.9 kb plasmid carries among others genes encoding the following proteins: ParB, Mpr4, VirB2, VirB4, VirB6, VirB8, VirB9, VitB10, VirB11, and VirD4 (**Figure 4C**).

### 3.4 Antimicrobial susceptibility testing and association with antibiotic resistance genes

Phenotypic resistance testing of *A. butzleri* isolates faces the difficulty that no clinical breakpoints are specified and therefore new epidemiological cut-off (Ecoff) values must be defined based on the frequency distribution of inhibition zone diameters taking the presence or absence of ARGs into account. Using ResFinder, 13 and 11 antibiotic resistance genes were detected in the multidrug resistant *A. butzleri* isolates P1100 and P1200, respectively. The aminoglycoside adenyltransferase *aadD* gene associated with kanamycin resistance and the *tetL* gene encoding a tetracycline:cation symporter, were present in P1100 but were absent in P1200. In both genome sequences, the tetracycline resistance genes *tetA* and *tetM*, the beta-lactamase *bla*_OXA-464_, and the macrolide resistance genes *macA*, *macB*, *tolC*, and *ermB* were found. Furthermore, the two isolates were found to be positive for streptomycin adenylyltransferase gene *str*, the chloramphenicol *O*-acetyltransferase gene *cat*, the streptomycin resistance associated gene of the aminoglycoside nucleotidyltransferase ANT(6)-Ia *ant(6)-Ia*, and the gentamicin resistance associated gene of the 3-aminoglycoside *O*-phosphotransferase type IIIa Aph(3)-IIIa *aph(3)-IIIa*. The ARG distribution (**Figure 1**) also indicated multidrug resistance in the *A. butzleri* isolates P1099 and P1167.

**Figure 5:**
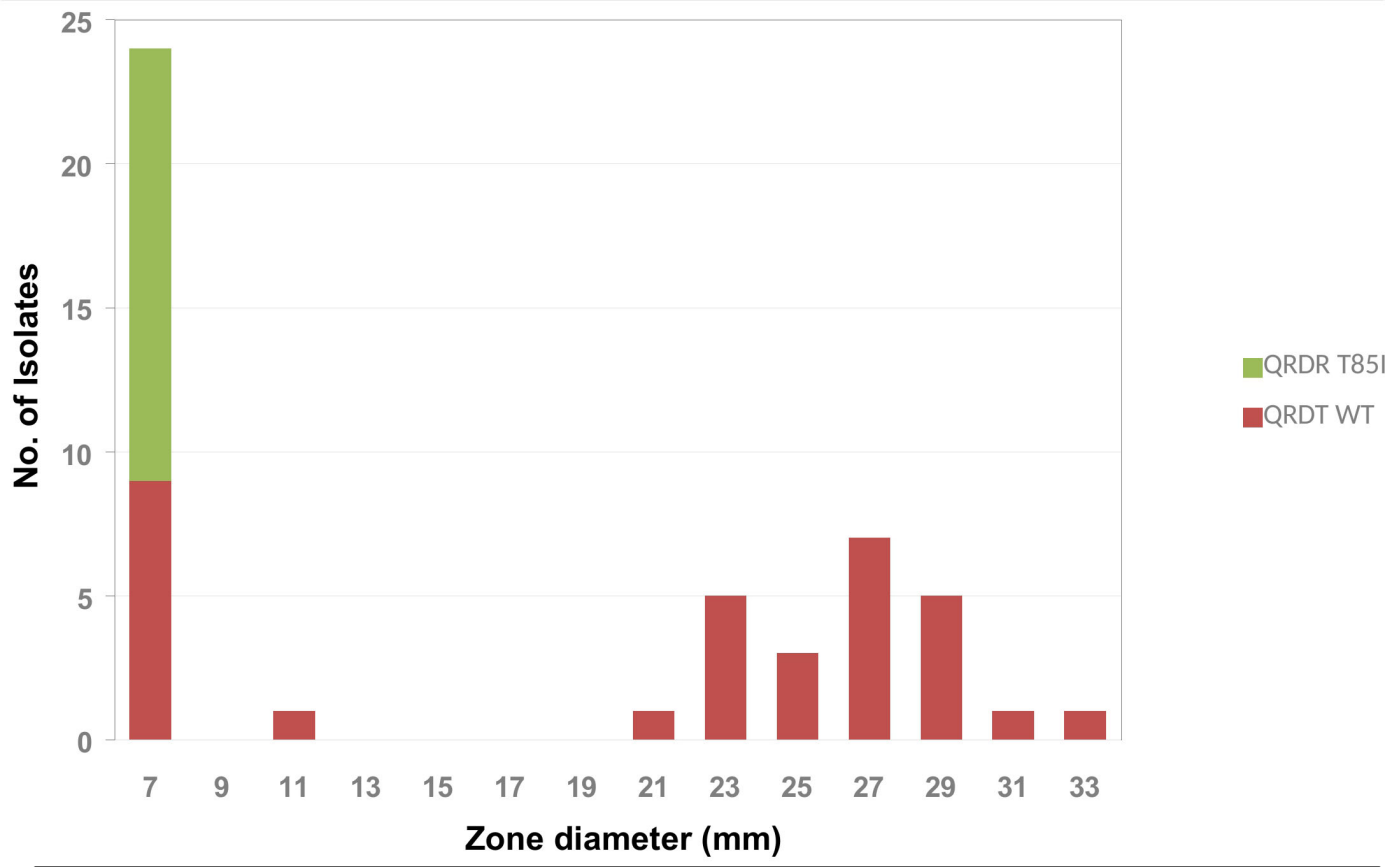
Frequency distribution of the T85I point mutation in the quinolone resistance determining region (QRDR) by ciprofloxacin inhibition zone diameter within *A. butzleri* isolates tested.

The amino acid substitution T85I in the QRDR, associated with quinolone resistance, was detected in 31.25% (15/48) of the isolates (**Figure 1**). Although both genome sequenced isolates were phenotypically resistant to ciprofloxacin, the amino acid substitution T85I was detected only in P1200 while P1100 showed the wild type amino acid sequence. The correlation of the inhibition zone size for ciprofloxacin with the presence of the amino acid substitution T85I in the QRDR showed that in addition to the 15 T85I-positive isolates, 10 isolates had a small (≤ 11 mm) inhibition zone diameter for ciprofloxacin (**Figure 5**). Consequently, there seems to be at least one further quinolone resistance determinant besides the amino acid substitution T85I in the QRDR. The quinolone susceptible *A. butzleri* population showed an inhibition zone diameter of ≥ 21 mm. Since no clinical breakpoints exist for *A. butzleri*, an epidemiological cut off at < 13 mm could be defined for ciprofloxacin.

**Figure 6:**
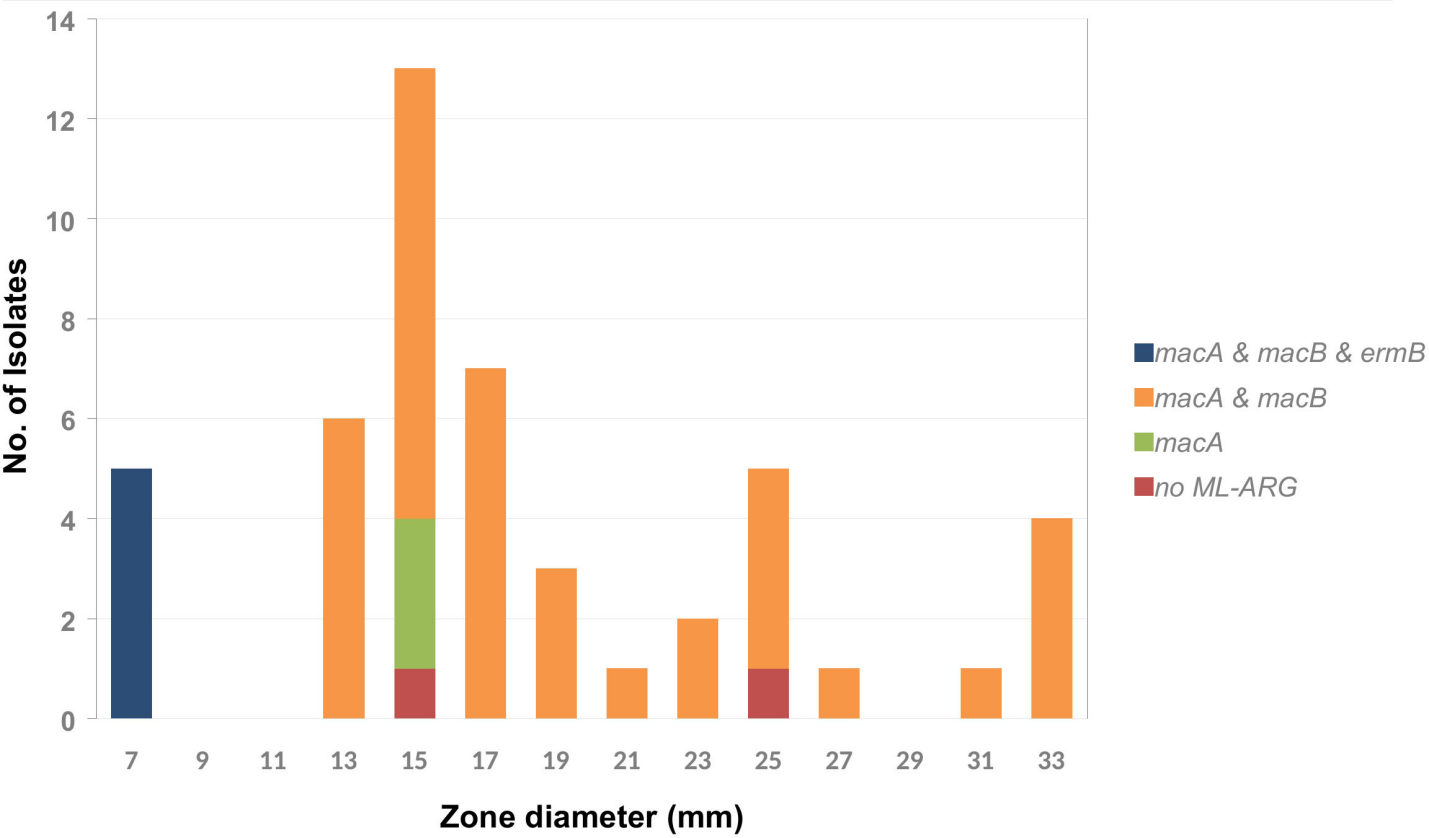
Frequency distribution of the macrolide resistance associated genes *macA*, *macB*, and *ermB* by erythromycin inhibition zone diameter within the tested *A. butzleri* isolates. The gene *tolC* occurs in 100% of the isolates of the tested collection and is therefore not included in the diagram.

The genes *macA*, *macB*, and *tolC* encode the components of an efflux pump associated with macrolide resistance occurred frequently with a few exceptions. As shown in **Figures 1**, **6**, there are five isolates that were positive for *ermB* (encoding a 23S rRNA (adenine(2058)-*N*(6))-methyltransferase) with a phenotypic inhibition zone diameter for erythromycin < 7 mm. The remaining isolates showed an erythromycin inhibition zone of > 13 mm to a maximum of 33 mm. According to this an epidemiological cut off at < 9 mm could be defined for erythromycin. The trimeric efflux pump from the gene products of *macA*, *macB*, and *tolC* does not seem to mediate detectable macrolide resistance, at least *in vitro*.

**Figure 7:**
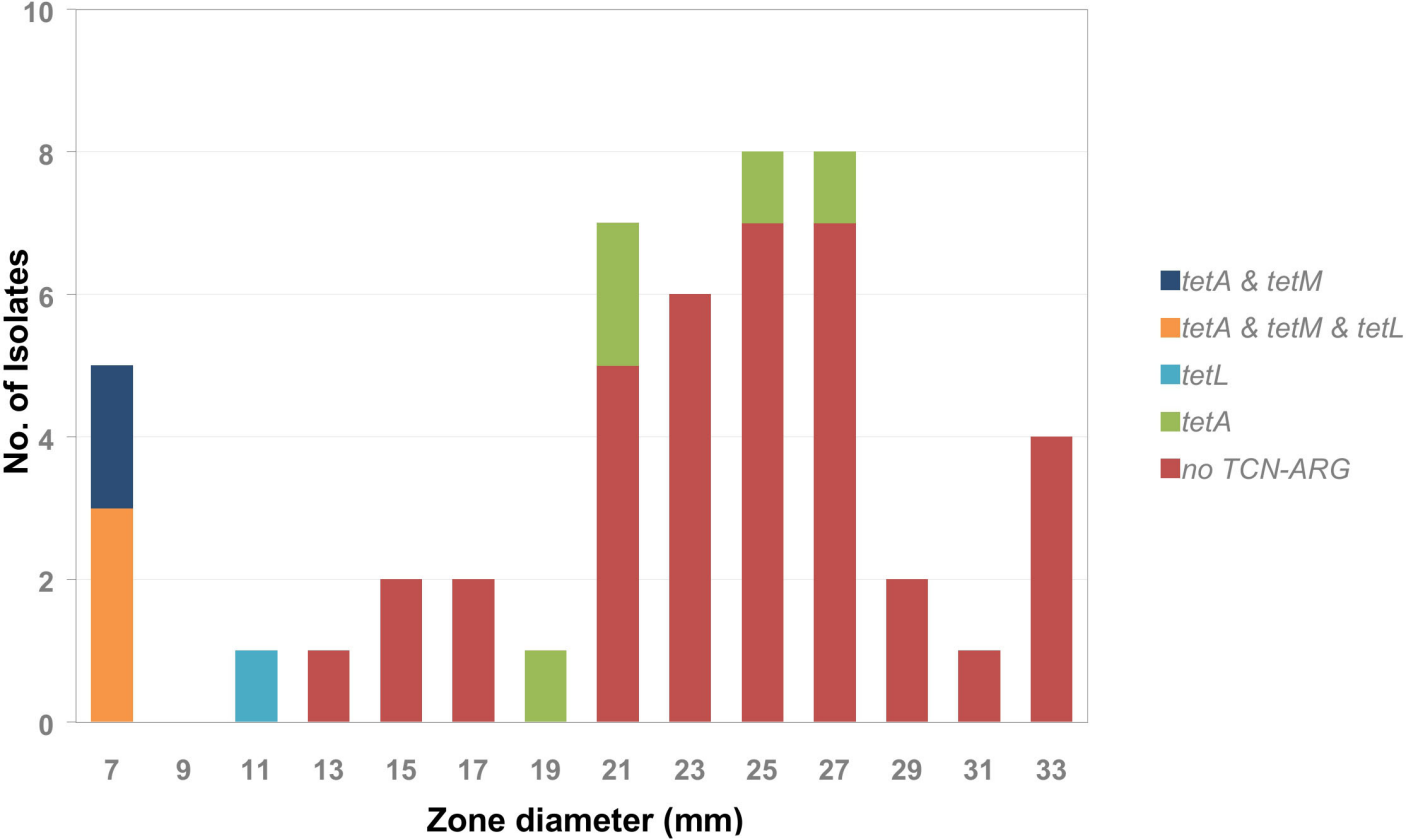
Frequency distribution of tetracycline resistance associated genes *tetA*, *tetM*, and *tetL* by tetracycline inhibition zone diameter within the tested *A. butzleri* isolates.

This was similar to what was found for the tetracycline (TCN) resistance genes (**Figure 7**). The isolates with an inhibition zone diameter of ≤ 7 mm were all *tetM*-positive. The *tetM* gene encodes a translation factor of the GTPase family that interacts directly with the ribosomal 70S subunit and thus inhibits tetracycline activity (Dönhöfer et al., 2012). All isolates with an inhibition zone diameter of ≤ 7 mm were also positive for *tetA* but only 60% were positive for *tetL* (encoding tetracycline:cation symporter transporter). A single isolate that was *tetL*-only positive had an inhibition zone diameter of 11 mm. In the group of isolates with an inhibition zone diameter between 13 mm and 33 mm the majority of isolates were negative for all tetracycline-resistance-associated ARGs. Only 12% were positive for *tetA*, which encodes a membrane bound tetracycline transporter, not sufficient for mediating tetracycline resistance. Consequently, an epidemiological cut off < 11 mm could be defined for tetracycline, which separates the *tetM* and/or *tetL* positive isolates from the tetracycline-ARG negative and *tetA* only positive isolates.

Three putative β-lactamases that confer resistance to β-lactams had been identified in the genome of *A. butzleri* RM4018 (Miller et al., 2007). Here the β-lactamase gene with locus tag Abu_1486 corresponded to the *bla*_OXA-464_ gene, which was also detected in both genomes. An examination of the ARG distribution in the *A. butzleri* test cohort (**Figure 1**) showed that the *bla*_OXA-464_ gene was present in 35 of 48 isolates and therewith it was almost ubiquitous, which correlates with penicillin and aminopenicillin resistance. Additionally, a homologue to the putative β-lactamase gene with locus tag Abu_1299 was detected in both genomes, P1100 and P1200. The putative β-lactamase gene with locus tag Abu_0578 could not be detected in the genome of P1200 nor in the genome of P1100. But also the genes *lrgA* and *lrgB*, which have been shown to influence penicillin tolerance in *Staphylococcus aureus* (Bayles, 2000; Groicher et al., 2000), were found in both *A. butzleri* genomes, P1100 and P1200, as well as in *A. butzleri* RM4018 (Miller et al., 2007). In phenotypic resistance testing, no discontinuous populations can be defined for gentamicin and ampicillin due to the limited number of isolates and thus no Ecoff can be determined. However, the distribution of the inhibition zone diameters suggests that all isolates are resistant to ampicillin (the majority of isolates have an inhibition zone diameter of 6 mm, which corresponds to the diameter of the test disc, i.e. no inhibition zone is visible), whereas no resistance to gentamicin was present (**Supplementary Table 1**).

To give an impression of how necessary it is to determine specific Ecoff values for a particular microbial species in correlation with the presence of ARGs, we would like to perform in the following the qualitative evaluation of the inhibition zone diameters based on the Ecoff values determined in this study in comparison to the qualitative results based on the EUCAST and CLSI inhibition zone diameter decision ranges for the closely related bacterial species *C. jejuni* or *C. coli*. According to the ciprofloxacin Ecoff, which was identified in our study, considering the T85I point mutation, 25 of 48 isolates (52.1%) were classified as unsusceptible (**Supplementary Table 1**). This is quite close to the qualitative evaluation according to CLSI. Here, 26 of 48 isolates (54.2%) were classified as resistant and 5 further as intermediate (10.4%). When evaluated according to EUCAST, 33 of 48 isolates (68.8%) are declared resistant, the remaining isolates are susceptible at increased exposure and none are susceptible. This appears to be very restrictive, as arcobacteriosis rarely needs to be treated with an increased dose of ciprofloxacin or with i.v. antibiosis. If erythromycin susceptibility is evaluated using the Ecoff value, which is based on the presence of the *ermB* gene, then only 5 of 48 isolates (10.4%) are classified as resistant. Using the CLSI decision ranges, 8 of 48 isolates (16.7%) would be classified as resistant and 16 (33.3%) as intermediate. Applying the *C. jejuni* EUCAST decision ranges, 35 of 48 isolates (72.9%) are erythromycin resistant and even 37 of 48 isolates (77.1%) when applying the *C. coli* decision ranges. Again, the application of the EUCAST decision ranges seems very restrictive and reflects very little on the presence of ARGs. When we applied the Ecoff value, which was based on the presence of the *tetM* gene, to assess tetracycline susceptibility, then 5 of 48 isolates (10.4%) were considered resistant. This is in strong contrast to the evaluation of the inhibition zone diameters according to the EUCAST decision ranges, indicating that 43 of 48 isolates (89.6%) should be considered resistant. The difference to the evaluation according to CLSI is not so large with 22 of 48 resistant isolates (45.8%), but here one must consider that there is also the category “intermediate” to which another 11 isolates (22.9%) belong, so that a total of 33 of 48 isolates (68.8%) were determined to be not completely susceptible according to CLSI. In summary, the evaluation according to CLSI as well as, and here to a greater extent, according to EUCAST, using the *C. jejuni* decision ranges, appears to be too restrictive and does not reflect the presence or absence of particular ARGs.

### 3.5 Virulence-associated genes

To determine the virulence of the two genome sequenced strains we investigated the presence of 16 putative virulence-associated factors previously described for *A. butzleri* (Miller et al., 2007) or *Campylobacter jejuni*.

Both genome sequences, P1100 and P1200, bear a homologue of the *Campylobacter* invasion antigen B gene (*ciaB*), a *cadF*-like outer membrane fibronectin-binding protein gene, a *cj1349*-like fibronectin/fibrinogen-binding protein, hemolysin A (*hlyA*), FtsJ-like RNA binding methyltransferase (*tlyA*), Phospholipase A1 (*pldA*), and the peptidoglycan biosynthesis protein gene (*mviN*). The gene *irgA* for an outer membrane receptor for ferrienterochelin and colicins (TonB-dependent receptor) was also detected in both genomes.

The serine protease HtrA, which additionally has a chaperone function, has been demonstrated to be a virulence-associated factor of higher importance in *C. jejuni*. HtrA cleaves occludin and E-cadherin and thereby can open tight junctions. In consequence, *C. jejuni* can access the basolateral compartment of the intestinal epithelium, is able to enter the bloodstream and can reach the mesenteric lymph nodes (Backert et al., 2013). An *htrA* homologue was also detected in the two *A. butzleri* genomes analyzed.

The genes encoding the hemagglutinin like protein (*hecA*/*hpmA*) and HecB family hemolysin secretion/activation protein (*hecB*), catecholate siderophore esterase (*iroE*), cytolethal distending toxin (*cdtABC*), gamma-glutamyltranspeptidase (*ggt*), dimethylsulfoxide (DMSO) reductase system (*dmsA*/*dmsB*) were absent in both genomes.

### 3.6 Lipooligosaccharide biosynthesis locus

The lipooligosaccharide biosynthesis locus (LOS-locus) is one of the genetically more variable regions in *Arcobacter* and *Campylobacter* species. In *A. butzleri* P1100 the LOS-locus extends over 28,470 bp (locus location: 383,853-412,323) and includes 30 ORFs, whereas in *A. butzleri* P1200 this locus extends over 31,059 bp (locus location: 1,592,138-1,623,197) and includes 31 ORFs. In both *A. butzleri* genomes 19 common genes are found in the LOS locus, flanked by the lipopolysaccharide heptosyltransferase I gene (*waaC*) and the D-glycero-D-manno-heptose 1,7-bisphosphate phosphatase gene. The differences between the two genomes were that P1100 contained additional genes for an UDP-4-amino-4-deoxy-L-arabinose–oxoglutarate aminotransferase, an *O*-acetyltransferase, and the glycosyltransferase WavQ, whereas the genome of P1200 contained additional genes for the dTDP-rhamnosyl transferase RfbF, the capsular polysaccharide repeating-unit polymerase CpsH, a galactoside *O*-acetyltransferase, and a phosphoglycerol transferase I. Moreover, there were also three copies of the poly(beta-D-mannuronate) *O*-acetylase gene in P1200. In both *A. butzleri* genomes, no sialyltransferase *cstII*/*cstIII* homologous genes were detected. Furthermore, no genes homologous to the CMP-Neu5Ac synthetase *neuA*, sialic acid synthase *neuB* and GlcNAc-6-phosphate 2-epimerase *neuC* were found in either genome. The enzymes *cstII*/*III* and *neuABC* have been shown to be a prerequisite for a sialic acid containing LOS, associated with increased invasiveness (Louwen et al., 2008) and induction of the Guillain-Barré-syndrome (GBS) in *C. jejuni* (Louwen et al., 2013). BLAST search in GenBank showed the absence of *cstII*/*III* homologous genes, while the genes homologous to *neuA*, *neuB*, and *neuC* in the genome of *Arcobacter peruensis* strain PSE-93 (GenBank: CP032363.1) and homologous to *neuB* and *neuC* were also found in the genome of *A. butzleri* 7h1h (GenBank: CP006615.1). Based on these findings, the development of GBS as a post-infectious sequel after *Arcobacter*-associated gastroenteritis is unlikely.

### 3.7 *N*-linked flagellar glycosylation genes

In *C. jejuni* and *C. coli* a *N*-linked flagellar glycosylation locus can be found directly downstream of the LOS-locus (Zautner et al., 2015). This *N*-linked flagellar glycosylation locus is flanked by an UDP-*N*-acetylglucosamine 4,6-dehydratase and a phospholipid-lipopolysaccharide ABC transporter gene e.g. in *C. coli* BfR-CA-9557 (Zautner et al., 2015). This locus does not exist in *A. butzleri*; rather, the individual genes for *N*-linked flagellar glycosylation are scattered over the entire genome. Nevertheless, the two flanking genes encoding an UDP-*N*-acetylglucosamine 4,6-dehydratase and a phospholipid-lipopolysaccharide ABC transporter were present in both *A. butzleri* genomes, P1100 and P1200. In addition, four further genes in both genomes are associated with *N*-linked flagellar glycosylation: *pglE* encoding an bacillosamine/legionaminic acid biosynthesis aminotransferase, *pglG* encoding a *N*-linked glycosylation glycosyltransferase, a gene encoding an UDP-glucose 4-epimerase (in 2 copies in P1200), and an UDP-*N*-acetylglucosamine 4,6-dehydratase gene (in four copies in P1200). In *A. butzleri* P1200 (but not in P1100) a 4-amino-6-deoxy-N-Acetyl-D-hexosaminyl-acetyltransferase gene was detected having a homologue in the *N*-linked flagellar glycosylation locus cluster of *C. coli* BfR-CA-9557. Furthermore, the P1200 genome contained a gene encoding an undecaprenyl-phosphate *N*-acetylglucosaminyl 1-phosphate transferase that was absent in *A. butzleri* P1100.

### 3.8 *O*-Antigen locus, capsular polysaccharide gene locus and CRISPR elements

Miller and colleagues hypothesized that *A. butzleri* RM4018 is most likely to produce an *O*-antigen but not a capsule as found in e.g. *C. jejuni* or *C. coli*. In *A. butzleri* RM4018 a locus has been identified that contains 2 copies of *wbpG*, *hisH*, and *hisF*, which are also present in the *O*-antigen locus of *Pseudomonas aeruginosa* (Miller et al., 2007). In contrast to RM4018, no homologues to *wbpG*, *hisF*, *O*-antigen acyltransferase gene, *pglJ*, or *asnB* were found in the genomes of *A. butzleri* P1100 and P1200. Only *hisH* was present in *A. butzleri* P1100 but not P1200. Consequently, the production of an *O*-antigen by both *A. butzleri* isolates is unlikely. Also, no cluster of conserved *kps* capsular genes was detected in P1100 and P1200, hence it is likely that as a result no capsule can be formed. Additionally, in both *A. butzleri* genome sequences no CRISPR elements were found.

### 3.9 Limitations of the study

There were few limitations of the presented study that need to be taken into consideration when interpreting our results. First, the sample size and thus the number of bacterial isolates grown was comparatively small. Second, samples and bacterial isolates originate only from one urban area, and the samples were predominantly taken from chicken meat originating from small farms. In consequence, our results may not be representative of all poultry meat consumed in Ghana, and accordingly also not for non-poultry meat. Therefore, the Ecoff values determined may have locally limited lower and upper bounds and variance that may not be globally applicable. Due to financial limitations it was not possible to sequence the whole genomes of all isolates. For this reason, we focused on two multidrug-resistant isolates sequenced by SMRT sequencing. Detection of ARGs in the majority of isolates was based on PCR with primers derived from these two genome sequences. Thus, negative results could be false negatives due to mutations in the primer binding site or a non-specific primer binding could lead to false positive results.

### 3.10 Conclusions

Because *Arcobacter* ssp. are not routinely detected in diarrheal diseases, their role in human disease is underestimated. Due to higher temperature conditions, its role in infections should be considered much higher in Africa than in temperate climates due to the the comparatively higher detection rates in Africa (Samie et al., 2007; Brückner et al., 2020). Here, the poultry intestine represents the main habitat where potential chains of infection can start. Due to the increased use of antibiotics in animal husbandry in Africa, there is a potential risk of the establishment of multi-resistant strains in poultry flocks, which can then be passed on to humans *via* food or contaminated water sources. In our study, we showed that these multidrug-resistant strains were already present in poultry flocks and had characterized two of these isolates by whole-genome sequencing in detail. We could show that most of the resistance genes are located on plasmids, but they can also integrate into the chromosome *via* mobile genetic elements (MGEs). Thus, both horizontal and vertical transmission of antibiotic resistance genes (ARGs) is possible. According to our resistance test results and determined Ecoff values, we would recommend erythromycin or tetracycline for the therapy of arcobacteriosis. Ciprofloxacin, with a resistance rate of about 50%, seems to have only limited applicability for therapy, at least in Ghana. Compared to *C. jejuni* or *C. coli*, the virulence factor gene set in the two isolates examined was lower. Also, the potential for inducing postinfectious sequelae such as GBS or reactive arthritis appeared to be much lower in the two genome sequenced isolates due to the LOS without sialic acid. It should be noted, that the typed isolates originate from poultry, and the virulence factor profile may be more complex in isolates originating from symptomatic patients. The combination of a more virulent subset of *A. butzleri* strains and the multi-resistant ARGs has the potential to cause difficulties in the future for both human and poultry treatment.

## Data availability statement

The datasets presented in this study can be found in online repositories. The names of the repository/repositories and accession number(s) can be found below: https://www.ncbi.nlm.nih.gov/genbank/, CP104378 https://www.ncbi.nlm.nih.gov/genbank/, CP104379 https://www.ncbi.nlm.nih.gov/genbank/, CP104380 https://www.ncbi.nlm.nih.gov/genbank/, CP104332 https://www.ncbi.nlm.nih.gov/genbank/, CP104333.

## Author contributions

AZ, JM and DD conceived the study idea, organized the bacterial isolate collection, and drafted the manuscript. KB and CA collected the isolates, performed species identification and susceptibility testing. JF performed QRDR amplification and sequencing. TR and JO performed whole genome sequencing and genome annotation. AK organized acquisition of funds. The further bioinformatic analyses were carried out by TR, AD and AZ. All authors contributed to the article and approved the submitted version.
